# D-Chiro-Inositol Improves Sperm Mitochondrial Membrane Potential: In Vitro Evidence

**DOI:** 10.3390/jcm9051373

**Published:** 2020-05-07

**Authors:** Rosita A. Condorelli, Federica Barbagallo, Aldo E. Calogero, Rossella Cannarella, Andrea Crafa, Sandro La Vignera

**Affiliations:** Department of Clinical and Experimental Medicine, University of Catania, 95123 Catania, Italy; federica.barbagallo11@gmail.com (F.B.); acaloger@unict.it (A.E.C.); rossella.cannarella@phd.unict.it (R.C.); crafa.andrea@outlook.it (A.C.); sandrolavignera@unict.it (S.L.V.)

**Keywords:** D-chiro-inositol, male infertility, sperm mitochondrial membrane potential

## Abstract

The use of inositols in endocrinological clinical practice is increasingly widespread. Most of the existing evidence concerns myoinositol (MYO), the most abundant form in nature, especially in women with polycystic ovarian syndrome. We have previously shown that MYO increases sperm motility in patients with asthenozoospermia by the increase of sperm mitochondrial membrane potential (MMP), a biofunctional sperm parameter closely associated to sperm motility. The aim of this study was to evaluate the effects of D-chiro-inositol (DCI), another biologically active isoform of inositols, on sperm MMP, as data on this matter has never been released so far. To accomplish this, semen samples from 15 patients with asthenozoospermia and 15 healthy normozoospermic men were incubated with increasing concentrations of DCI (0, 75, and 750 µg/mL) or phosphate buffer saline for 30 min. Incubation with DCI significantly improved sperm MMP at lower concentrations, and with shorter incubation length than those used in our similar MYO studies. In conclusion, these findings indicate that DCI positively impacts on sperm mitochondrial function in vitro. Studies aimed at assessing the role of DCI in the treatment of asthenozoospermia in-vivo are warranted.

## 1. Introduction

The use of inositol is widespread in endocrinologic clinical practice, especially for its insulin-sensitizing activity, largely exploited for the treatment of polycystic ovary syndrome (PCOS) [1]. Moreover, inositol antioxidant action was reported to be useful in autoimmune thyroid diseases [2], while there was limited evidence of the use of myo-inositol (MYO) in patients with asthenozoospermia.

Inositols are cyclic carbohydrates with six hydroxyl groups (C_6_H_12_O_6_). MYO is the most abundant form in nature, but it is only one of the nine stereoisomeric forms of inositols (named scyllo- muco-, epi-, neo-, allo-, cis-, D-chiro-, and L-chiro-inositols) [3]. MYO can be converted into D-chiro-inositol (DCI) by a specific epimerase stimulated by insulin. Both MYO and DCI are components of cell membrane phospholipids, with a structural and functional role. Indeed, cell membrane phospholipids are the source of inositol triphosphate (IP3), diacylglycerol (DAG), and inositolphosphoglycans (IPG) that act as second messengers of many metabolic pathways. IP3 regulates the activity of different hormones, including follicle-stimulating hormone (FSH) and thyroid-stimulating hormone (TSH). Moreover, IP3 binds to receptors on mitochondria and endoplasmic reticulum, inducing calcium release from internal stores, which activates protein kinase C and mediates cellular responses. This process is also responsible for acrosome reaction, essential for allowing spermatozoa to penetrate the zona pellucida (ZP), and therefore for fertilization [4]. In addition, intracellular calcium increase leads to a concomitant increase of calcium into the flagellum, thus prompting myotubule contraction, flagella movements [5], and an increase of calcium into the mitochondria; which, in turn, enhances oxidative metabolism and ATP production [6], the latter supporting the flagellum energy demand.

Both MYO and DCI can be transformed in inositolphosphoglycans (IPGs), MYO-IPG and DCI-IPG respectively, which are important second messengers involved in insulin action. However, they have different function. MYO-IPG stimulates glucose transporter type 4 (GLUT4) translocation to the cell membrane, increasing cell uptake of glucose, whereas DCI-IPG mediates glycogen synthesis [4]. In addition, MYO and DCI have a specific ratio between them in the different tissues [7]. High levels of DCI are found in the cells responsible for glycogen storage (such as liver, muscles, and fat cells), whereas MYO is found more abundantly in tissues with high glucose consumption (such as the brain and heart). MYO/DCI plasma ratio is 40:1, and several studies have shown that this rate is the most effective for the treatment of PCOS [7].

We have shown that MYO increases sperm motility both in normozoospermic men and in patients with abnormal sperm parameters in vitro, by improving sperm mitochondrial membrane potential (MMP) in patients with oligo-astheno-teratozoospermia (OAT) [8,9]. In addition, a pharmacological response to MYO was reported in insulin resistant patients with asthenozoospermia and low MMP (so-called asthenozoospermia associated with a metabolic factor). However, a proportion of patients do not benefit from the administration of MYO alone, in terms of increased sperm motility [10,11,12]. The reasons for this poor efficacy are not well known. It can be hypothesized that a different absorption profile, a possible defect of epimerization into DCI and/or the need to associate DCI to MYO (as well as the addition of other trace elements), may be necessary for the correct sperm maturation, thus implementing therapeutic efficacy.

Based on these premises, this study was undertaken to evaluate the in vitro effects of DCI on sperm mitochondrial function, considering a biofunctional parameter capable of predicting alterations in sperm motility [13] in patients with astenozoospermia and in normozoospermic men.

## 2. Patients and Methods

This protocol was approved by the Institutional Review Board (6/2019), and informed written consent was obtained from each man.

### 2.1. In Vitro Study

The study was conducted on 15 patients with asthenozoospermia and 15 healthy normozoospermic men (mean age of 25.0 ± 6.0 vs. 27.0 ± 4.0, with body mass index of 22.0 ± 1.5 vs. 20.5 ± 2.5, and mean HOMA values of 1.2 ± 0.3 vs. 1.6 ± 0.5). They did not have male accessory glands infection, systemic diseases, micro-orchidism (testicular volume <12 mL), cryptorchidism, or varicocele (any grade), and did not receive hormonal treatment in the last 12 months.

### 2.2. Sperm Preparation

Sperm samples were collected after 3–5 days of sexual abstinence. After liquefaction, sperm analysis was conducted according to the WHO 2010 criteria [14]. The remaining sample was used for flow cytometry analysis. For this purpose, aliquots containing 1 × 10^6^/mL of spermatozoa were prepared with phosphate buffered saline and centrifuged for 5 min at 5000× *g*.

### 2.3. Chemicals

D-Chiro was purchased from Amicogen, Jinjiu City, South Korea.

### 2.4. Experimental Design

Spermatozoa were incubated with increasing concentrations of DCI (0, 75, and 750 µg/mL) or phosphate buffer saline (the vehicle used to solubilize DCI) at 37 °C in a water-jacketed incubator under 5% CO_2_ atmosphere for 30 min. The concentrations of 75 and 750 µg/mL of DCI were chosen for all the experiments, according to a previous study of MYO and known inositol pharmacokinetics [8]. At the end of incubation, spermatozoa were evaluated for MMP by flow cytometry.

### 2.5. Flow Cytometric Analysis

Flow cytometric analysis was performed using flow cytometer CytoFLEX (Beckman Coulter Life Science, Milan, Italy) equipped with two argon lasers and six total fluorescence channels (four 488 nm and two 638 nm). We used the FL1 detectors for green (525 nm), FL2 for orange (585 nm), and FL3 for red (620 nm) fluorescence; 100,000 events (low velocity) were measured for each sample and analyzed by the software, CytExpert 1.2.

### 2.6. Evaluation of the Mitochondrial Membrane Potential

MMP was evaluated by a lipophilic probe 5,5’,6,6’-tetrachloro-1,1’,3,3’tetraethyl-benzimidazolylcarbocyanine iodide (JC-1, DBA s.r.l, Milan, Italy) which is able to selectively penetrate into mitochondria.

Briefly, an aliquot containing 1 × 10^6^/mL of spermatozoa was incubated with JC-1 in the dark for 10 min at 37 °C. At the end of the incubation period, the cells were washed in PBS and analyzed. JC-1 exists in monomeric form, emitting at 527 nm, but it is able to form aggregates emitting at 590 nm. Therefore, the fluorescence changes reversibly from green to orange when the mitochondrial membrane becomes more polarized. In viable cells with normal membrane potential, JC-1 is in the mitochondrial membrane in form of aggregates emitting an orange fluorescence; while in the cells with low membrane potential, it remains in the cytoplasm in a monomeric form and has a green fluorescence.

### 2.7. Statistical Analysis

Data are reported as mean ± SEM throughout the study. The normal distribution of each variable was evaluated with the Shapiro–Wilks test. Statistical analysis was performed by one-way analysis of analysis of variance (ANOVA), followed by Duncan’s Multiple Range test or Student’s *t* test, as appropriate. The statistical evaluation of the percentage of patients with normal sperm motility at the end of the treatment was conducted by Chi square analysis. The software SPSS 23.0 for Windows (SPSS Inc., Chicago, IL, USA) was used. A *p*-value lower than 0.05 was considered statistically significant.

## 3. Results

### In Vitro Study

Conventional sperm parameters and percentage of spermatozoa with high mitochondrial membrane potential (H-MMP) or low mitochondrial membrane potential (L-MMP) in normozoospermic men and in patients with asthenozoospermia patients enrolled in the in vitro study are listed in Table 1.

**Figure 1:**
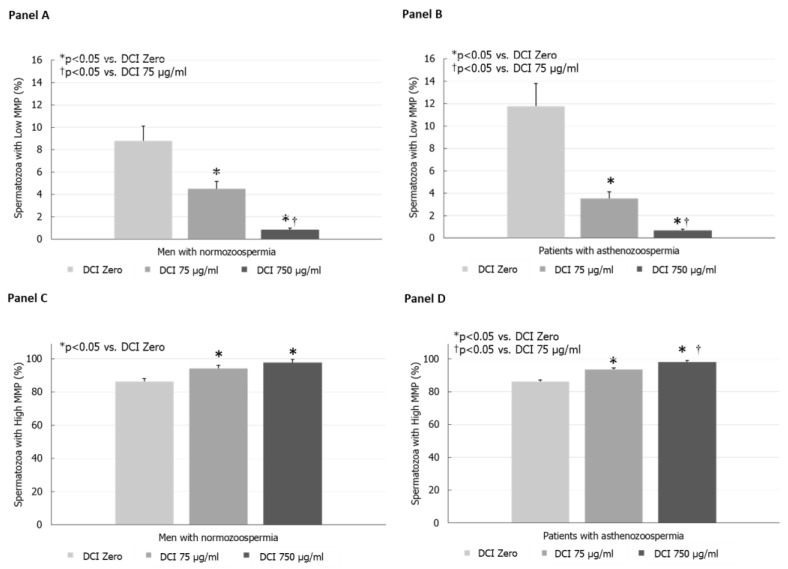
(**Panel A**) Effects of increasing concentrations of D-Chiro-inositol (DCI) on the percentage of spermatozoa with low mitochondrial membrane potential (L-MMP) in normozoospermic men. (**Panel B**) Effects of graded concentrations of DCI on the percentage of spermatozoa with high L-MMP in asthenozoospermic patients. (**Panel C**) Effects of graded concentrations of DCI on the percentage of spermatozoa with high mitochondrial membrane potential (MMP) in normozoospermic men. (**Panel D**) Effects of graded concentrations of DCI on the percentage of spermatozoa with high H-MMP in patients with asthenozoospermia.

DCI in vitro decreased the percentage of spermatozoa with L-MMP in both normozoospermic men (Figure 1, panel A) and patients with asthenozoospermia (Figure 1, panel B) in a concentration-dependent manner (*p* < 0.005, ANOVA). The effects of DCI, at both the concentrations of 75 and 750 µg/mL, were statistically significant compared with DCI zero (*p* < 0.05, ANOVA followed by Duncan’s test), and became higher at a concentration of 750 µg/mL compared with 75 µg/mL (*p* < 0.05, ANOVA followed by Duncan’s test).

The percentage of spermatozoa with H-MMP increased significantly after incubation with 75 and 750 µg/mL DCI (*p* < 0.05 vs. DCI zero, ANOVA followed by Duncan’s test) in men with normozoospermia. No statistically significant difference was found between the concentrations of 75 µg/mL and 750 µg/mL (Figure 1, panel C).

In patients with asthenozoospermia, H-MMP increased in a concentration-dependent fashion after incubation with 75 and 750 µg/mL of DCI (*p* < 0.05 vs. DCI zero, ANOVA followed by Duncan’s test). At a concentration of 750 µg/mL, DCI was statistically more effective than 75 µg/mL (*p* < 0.05, ANOVA followed by Duncan’s test) (Figure 1, panel D).

## 4. Discussion

The results of this in vitro study showed that DCI ameliorates sperm mitochondrial function at lower concentrations, and with a shorter length of incubation than those required for MYO in a similar experimental design [9].

The mitochondrion represents an organelle of pivotal importance for sperm motility and fertilization. Accordingly, it provides energy for sperm motility by glycolysis and oxidative phosphorylation. The evaluation of MMP gives information on sperm mitochondrial function. A healthy mitochondrion is capable of fully supporting sperm energy demand [13].

We report here, for the first time, that DCI has a beneficial effect on sperm mitochondrial function. It decreased the in vitro percentage of spermatozoa with L-MMP in a concentration-dependent manner, both in normozoospermic men and in patients with asthenozoospermia. Accordingly, the percentage of spermatozoa with H-MMP increased in a concentration-dependent fashion after incubation with DCI. We also showed that MYO is able to ameliorate the mitochondrial function of human spermatozoa in an in vitro experimental model, similar to that used for DCI in the present study. However, it should be pointed out that MYO was efficacious in decreasing the percentage of spermatozoa with L-MMP at the concentration of 2 mg/mL after 2 h of incubation [9]. DCI was effective after 30 min of incubation and suppressed the percentage of spermatozoa with L-MMP by about 90%. MMP evaluation is a very useful test for evaluating sperm mitochondrial function, where ATP production takes place. Spermatozoa with L-MMP have lower sperm motility, because flagellar movement is ATP-dependent [15]. Therefore, a small percentage of spermatozoa with L-MMP was suggestive of a seminal fluid of “better quality” and resulted in a higher fertilization rate after in vitro fertilization [15].

The beneficial effects of inositols on sperm motility and mitochondrial function can be due to their many actions: insulin-sensitizing properties, antioxidant and prokinetic activity, and hormonal regulatory effects.

Glucose metabolism is fundamental for spermatogenesis, and spermatozoa have glucose transporters (GLUT) along their entire surface. The latter uses glucose and fructose to generate energy for their many processes (capacitation, acrosome reaction, motility, etc.), whereas the other germ cells use lactate provided by Sertoli cells during spermatogenesis [16]. The conversion of glucose into pyruvate and then lactate is regulated by FSH and insulin, which favors the passage of lactate into the intratubular fluid to be subsequently internalized by germ cells [16]. Therefore, in the presence of insulin-resistance, the insulin-sensitizing properties of inositols could play a relevant role on sperm function.

It is possible to hypothesize an incomplete insulin-sensitizing action of MYO alone due to a defect in tissue epimerization or the necessary synergistic action with its biologically active form (DCI). However, there is no evidence in literature to date on testicular epimerase capable of converting MYO into DCI.

DCI accelerates glucose uptake and activates glycogen synthase in muscle biopsies beyond that of maximal insulin stimulation [17]. Recent clinical evidence showed that MYO (1100 mg) and DCI (27.6 mg) acutely lowered insulin response after glucose intake in obese insulin-resistant male children with hyperinsulinemia [18]. In previous studies, we documented the long-term alterations of the testicular function of adolescents with insulin-resistance, where sperm parameter abnormalities found in adulthood represented long-term complications of insulin-resistance and hyperinsulinemia that can be improved by using insulin sensitizers [19].

In conclusion, the results of the present study, although preliminary, suggest that DCI can be candidate compounds for the treatment of asthenozoospermia associated with altered sperm mitochondrial function [11]. However, further clinical studies and in vitro data are needed.

## 5. Main limitations of the Study

Some limitations can be recognized in this study. Firstly, even if the study of MMP represents a predictive parameter of suitable motility [13], it cannot resume the overall mitochondrial function alone. Accordingly, an extremely high MMP could lead to increased production of reactive oxygen species (ROS) and worsen sperm motility. Thus, further in vitro studies, with larger sets of patients, are needed to confirm the in vitro effects of DCI on sperm motility and to better highlight the molecular mechanisms underlining this process. This research represents a pilot and limited in vitro experiment, which could serve as a basis for future in vitro and in vivo studies. Secondly, the doses of DCI deserve to be standardized by further studies.

## Figures and Tables

**Table 1:** Conventional sperm parameters and percentage of spermatozoa with high or low mitochondrial membrane potential (MMP) in men with normozoospermia and patients with asthenozoospermia enrolled in the in vitro part of the present study.

	Men with Normozoospermia	Patients with Asthenozoospermia	*p*-Value
Volume (mL)	2.6 ± 0.26	2.3 ± 0.36	NS
Sperm concentration (10^6^/mL)	39.6 ± 5.3	25.7 ± 8	NS
Total sperm count (10^6^/ejaculate)	104.7 ± 18.6	48.7 ± 16.5	<0.05
Progressive motility (%)	33.1 ± 0.64	19.7 ± 1.87	<0.01
Total motility (%)	52 ± 3.5	41.2 ± 4.5	NS
Normal forms (%)	6.8 ± 1.2	8.0 ± 2.7	NS
Leukocytes (10^6^/mL)	0.5 ± 0.2	1.0 ± 0.2	NS
High MMP (%)	86.2 ± 3.1	86.2 ± 0.8	NS
Low MMP (%)	8.8 ± 1.4	11.8 ± 0.5	<0.05

Results are expressed as mean ± SEM. NS: not signifivant.
